# Mitochondrial DNA haplotypes induce differential patterns of DNA methylation that result in differential chromosomal gene expression patterns

**DOI:** 10.1038/cddiscovery.2017.62

**Published:** 2017-09-11

**Authors:** William T Lee, Xin Sun, Te-Sha Tsai, Jacqueline L Johnson, Jodee A Gould, Daniel J Garama, Daniel J Gough, Matthew McKenzie, Ian A Trounce, Justin C St. John

**Affiliations:** 1 Centre for Genetic Diseases, Hudson Institute of Medical Research, 27-31 Wright Street, Clayton, Victoria 3168, Australia; 2 Department of Molecular and Translational Science, Monash University, 27-31 Wright Street, Clayton, Victoria 3168, Australia; 3 Medical Genomic Facility, Monash Health Translational Precinct, 27-31 Wright Street, Clayton, Victoria 3168, Australia; 4 Centre for Cancer Research, Hudson Institute of Medical Research, 27-31 Wright Street, Clayton, Victoria 3168, Australia; 5 Centre for Eye Research Australia, Ophthalmology, University of Melbourne Department of Surgery, 32 Gisborne Street, East Melbourne, Victoria 3002, Australia

## Abstract

Mitochondrial DNA copy number is strictly regulated during development as naive cells differentiate into mature cells to ensure that specific cell types have sufficient copies of mitochondrial DNA to perform their specialised functions. Mitochondrial DNA haplotypes are defined as specific regions of mitochondrial DNA that cluster with other mitochondrial sequences to show the phylogenetic origins of maternal lineages. Mitochondrial DNA haplotypes are associated with a range of phenotypes and disease. To understand how mitochondrial DNA haplotypes induce these characteristics, we used four embryonic stem cell lines that have the same set of chromosomes but possess different mitochondrial DNA haplotypes. We show that mitochondrial DNA haplotypes influence changes in chromosomal gene expression and affinity for nuclear-encoded mitochondrial DNA replication factors to modulate mitochondrial DNA copy number, two events that act synchronously during differentiation. Global DNA methylation analysis showed that each haplotype induces distinct DNA methylation patterns, which, when modulated by DNA demethylation agents, resulted in skewed gene expression patterns that highlight the effectiveness of the new DNA methylation patterns established by each haplotype. The haplotypes differentially regulate *α*-ketoglutarate, a metabolite from the TCA cycle that modulates the TET family of proteins, which catalyse the transition from 5-methylcytosine, indicative of DNA methylation, to 5-hydroxymethylcytosine, indicative of DNA demethylation. Our outcomes show that mitochondrial DNA haplotypes differentially modulate chromosomal gene expression patterns of naive and differentiating cells by establishing mitochondrial DNA haplotype-specific DNA methylation patterns.

## Introduction

The murine mitochondrial genome (mtDNA) is a double-stranded, ~16.3 kb, circular genome.^1^ It encodes 13 proteins of the electron transfer chain, which generates the vast majority of cellular energy through oxidative phosphorylation (OXPHOS). Whilst the majority of the subunits of the electron transfer chain are encoded by the nuclear genome, each of the complexes, except for complex II, has one or more of its proteins encoded by mtDNA.^2^ mtDNA also encodes two rRNAs and 22 tRNAs and has one major non-coding region, the D-loop. The D-loop possesses two hypervariable regions that identify maternal relatives,^3^ and is the site of interaction for the nuclear-encoded transcription and replication factors.^4^

mtDNA copy number is strictly regulated during development and differentiation.^5^ The primordial germ cells possess ~200 copies of mtDNA,^6^ which exponentially increase during oogenesis until the mature, fertilisable oocyte has >150 000 copies.^7,8^ Following fertilisation, there is active reduction of mtDNA copy number until the blastocyst stage, the final stage of preimplantation development.^6,8^ Whilst the blastocyst’s outer ring of cells, the trophectodermal cells, replicate mtDNA as they differentiate into the trophectoderm,^8^ the inner cell mass cells, which form the embryo proper and are the source of embryonic stem (ES) cells, further reduce mtDNA copy number to establish the mtDNA set point.^8,9^

The mtDNA set point ensures that all naive (undifferentiated, pluripotent) cells maintain low mtDNA copy number, and, thus, use glycolysis to generate ATP.^10^ This promotes cellular proliferation to enable the embryo to generate a critical mass of cells for post-gastrulation development. Once differentiation is initiated, cells replicate their mtDNA in a cell-specific manner,^11^ which is mediated by the cell-specific DNA methylation of a CpG island in exon 2 of the catalytic subunit of the mtDNA-specific replication factor, DNA polymerase gamma (*PolgA*).^12^ Therefore, cells with a high requirement for ATP through OXPHOS, such as heart, muscle and neuronal cells, acquire high numbers of mtDNA copy, whilst cells with a lower requirement for ATP possess fewer copies of mtDNA and use glycolysis.^13^

mtDNA copy number is important to cellular fate. Altering mtDNA copy number in tumour cells can modulate chromosomal gene expression patterns, and promote differentiation.^11,14^ Likewise, mtDNA haplotypes can influence chromosomal gene expression patterns in ES cells^15^ and tumours.^14^ mtDNA haplotypes are defined by specific regions of mtDNA that identify the phylogenetic origins of maternal lineages.^16^ In a range of species, mtDNA haplotypes are associated with adaptation to warm and cold environments,^17^ predisposition to diseases of aging such as cancer,^18^ diabetes,^19^ Alzheimer’s^20^ and Parkinson’s,^21^ and fertility.^22,23^

We have investigated whether chromosomal gene expression patterns can be altered in a haplotype-specific manner due to modulation of global DNA methylation patterns. We assessed global patterns of hypo- and hypermethylation in four ES cell lines each possessing the same chromosomal genotype but different mtDNA genotypes, namely mtDNA divergent ES cell lines. We assessed their mtDNA replicative efficiency during differentiation and, using DNA demethylation agents, determined whether their DNA methylation patterns could be altered to modulate chromosomal gene expression patterns.

## Results

### Next-generation sequencing of mitochondrial genomes

**Figure 1:**
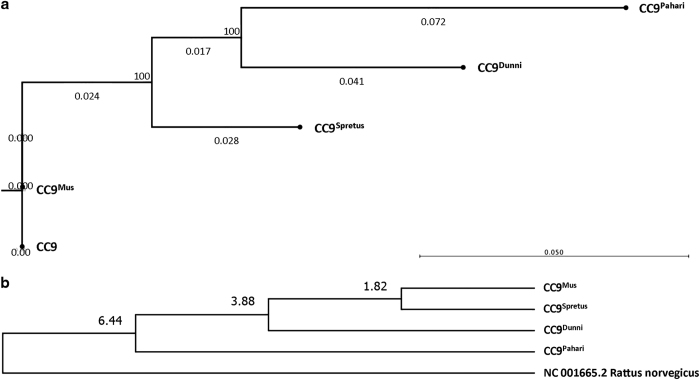
The phylogenetic relationship between the divergent mtDNA ES cell lines. (**a**) Phylogenetic clustering of mtDNA haplotypes from CC9^mus^, CC9^spretus^, CC9^pahari^ and CC9^dunni^ whole mitochondrial genome sequences. A Maximum Likelihood phylogenetic tree was constructed with the GTR model and Neighbor-Joining method with 1000 bootstrap replicates. Bootstrap values are expressed as a percentage; (**b**) Molecular Phylogenetic analysis by the Maximum Likelihood method. Time of divergence was estimated using the RelTime method. The estimated divergence time for *M. musculus* and *M. spretus* was 1.82 Mya; *M. musculus* and *M. dunni* was 3.88 Mya; and *M. musculus* and *M. pahari* was 6.44 Mya.

We sequenced the mitochondrial genomes of four mtDNA divergent mouse ES cell lines (CC9^mus^, CC9^spretus^, CC9^dunni^ and CC9^pahari^) generated from the fusion of enucleated cytoplasts of *Mus musculus*, *Mus spretus*, *Mus dunni* and *Mus pahari* cells to mitochondrial depleted *M. musculus* (CC9.3.1) ES cells; and the parental CC9.3.1 ES cell line to determine their genetic diversity. Figure 1a shows the phylogenetic representation of the four lines relative to the parental line. As the CC9^mus^ line possessed the same mtDNA genotype as the parental line, it was used to exclude bias resulting from generation of the cells. The degree of divergence between the CC9^mus^ and the CC9^spretus^ lines is 1.82 Mya, CC9^dunni^ is 3.88 Mya and CC9^pahari^ is 6.44 Mya (Figure 1b). Supplementary Table S1 shows the single-nucleotide polymorphisms amongst the haplotypes and Supplementary Table S2 the respective changes in amino-acid codons.

### Gene expression analysis

To determine whether mitochondrial haplotypes influence the differentiation potential of the four mtDNA divergent ES cell lines, each line was induced to undergo neural differentiation. We analysed cells by real-time PCR for expression of master regulators and endpoint markers of differentiation at 3, 12 and 21 days of differentiation. The nine genes included *Musashi1*, a neural precursor marker; *Nestin*, a primitive neuroepithelial marker; *Ncam1*, an immature neuronal committed progenitor marker; *Sox1*, a neuroectodermal marker; *Pax6*, an advanced neuronal precursor cell marker; *Tubb3*, indicative of newly differentiated neurons; *Map2a*, indicative of mature neurons; *Gfap*, indicative of mature astrocytes; and *Syp*, indicative of mature neurons with synaptic vesicles.

**Figure 2:**
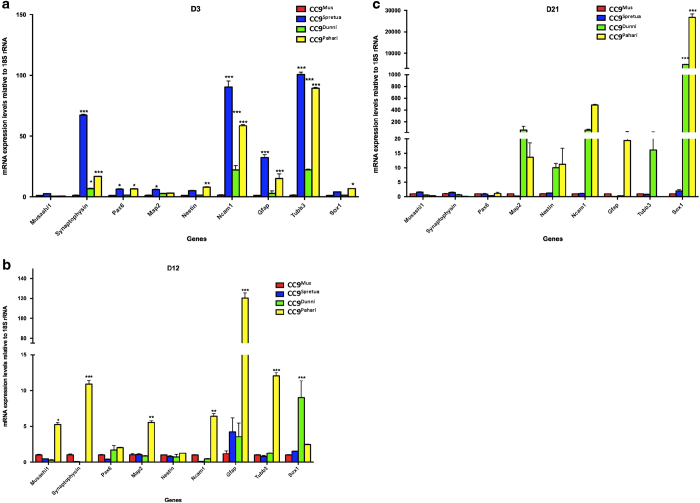
Gene expression during neural differentiation of divergent mtDNA ES lines. CC9^mus^, CC9^spretus^, CC9^dunni^ and CC9^pahari^ ES cells were induced to differentiate into neural lineages over 21 days and were assessed at days 3 (**a**), 12 (**b**) and 21 (**c**) for expression of *Musashi1*, *Synaptophysin*, *Pax6*, *Map2*, *Nestin*, *NCAM1*, *Gfap*, *Tubb3* and *Sox1* by real-time PCR. *P<0.05; **P<0.01; ***P<0.001.

Apart from Musashi1, on day 3, the lines showed discordant patterns of expression of the master regulators of neural differentiation (Figure 2a). Whilst there was the anticipated upregulation of *Pax6* with CC9^spretus^ and CC9^pahari^ cells being significantly different, *Ncam1* and *Tubb3* were significantly higher in each of the lines apart from CC9^mus^ cells. Both *Nestin* and *Sox1* also showed upregulation in CC9^pahari^ cells. For the endpoint markers, there was precocious expression of *Syp* in CC9^spretus^ cells and less so in CC9^dunni^ and CC9^pahari^ cells with a similar pattern for *Gfap* in CC9^spretus^ and CC9^pahari^ cells whilst only CC9^spretus^ was upregulated for *Map2a*. On day 12, CC9^pahari^ cells had very high levels of expression for each of the genes, including precocious expression of the endpoint markers *Map2a*, *Gfap* and *Syp* (Figure 2b). On day 21, CC9^mus^ and CC9^spretus^ cells regulated expression at similar levels but there were significant increases in CC9^dunni^ and CC9^pahari^ cells for *Sox1*, and nonsignificant increases for *Map2a*, *Nestin*, *Ncam1*, *Gfap* (CC9^pahari^ only), *Tubb3* (CC9^dunni^ only) and *Sox1. *Consequently, there were discordant patterns of neural gene expression during differentiation and at endpoint based on a cell’s mtDNA haplotype, which were exaggerated as divergence increased (Figure 2c).

### mtDNA replicative efficiency

**Figure 3:**
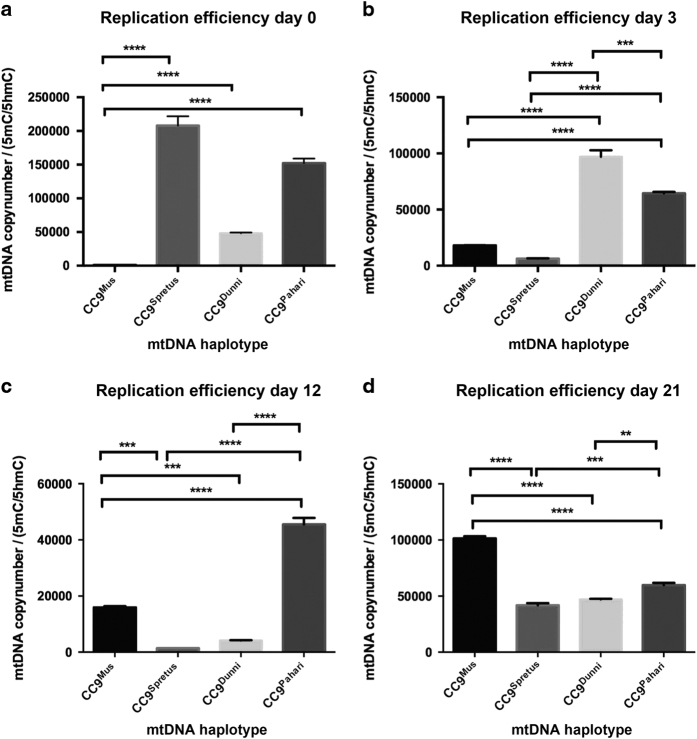
Replicative efficiency of divergent mtDNA ES lines. CC9^mus^, CC9^spretus^, CC9^dunni^ and CC9^pahari^ ES cells were induced to differentiate into neural lineages over 21 days and were assessed at days 0 (**a**), 3 (**b**), 12 (**c**) and 21 (**d**) of differentiation for mtDNA copy number and ratios of 5mC and 5hmC. MtDNA copy number was assessed by real-time PCR. Levels of enrichment for 5mC and 5hmC were assessed by MeDIP using antibodies against 5mC and 5hmC, and real-time PCR across exon 2 of PolgA. The data are expressed as a ratio of mtDNA copy against 5mC/5hmC, where 5mC and 5hmC are indicative of DNA methylation and DNA demethylation, respectively. **P<0.01; ***P<0.001; ****P<0.0001.

As the divergent ES cell lines showed discordant patterns of expression during neural differentiation, we determined their capacity to regulate mtDNA copy number. We assessed mtDNA copy number per cell for each line and expressed this value as a function of the ratio of 5-methylcytosine (5mC—DNA methylation) to 5-hydroxymethylcytosine (5hmC—DNA demethylation) within exon 2 of *PolgA*, that is, their mtDNA replicative efficiencies. Undifferentiated CC9^mus^ cells exhibited low mtDNA replicative efficiency (Figure 3a), as expected for undifferentiated murine ES cells.^9,12^ However, efficiencies were significantly higher for the other lines. On day 3 of differentiation (Figure 3b), there was a slight increase in mtDNA replicative efficiency for CC9^mus^ cells, indicative of increased mtDNA copy number and the onset of differentiation,^9^ whilst CC9^spretus^ cells returned to very low levels. However, CC9^dunni^ and CC9^pahari^ cells maintained significantly high efficiencies.

On day 12 (Figure 3c), CC9^mus^ cells further increased their replicative efficiency in synchrony with a more differentiated state. However, CC9^spretus^ and CC9^dunni^ cells had significantly lower and CC9^pahari^ cells significantly higher efficiencies. On day 21 (Figure 3d), CC9^mus^ cells increased their mtDNA replicative efficiency, whilst the other lines had significantly lower efficiencies. Consequently, only CC9^mus^ cells exhibited the potential to replicate mtDNA copy synchronously during differentiation.

### Levels of enrichment for POLGA, ESRRB and TFAM

**Figure 4:**
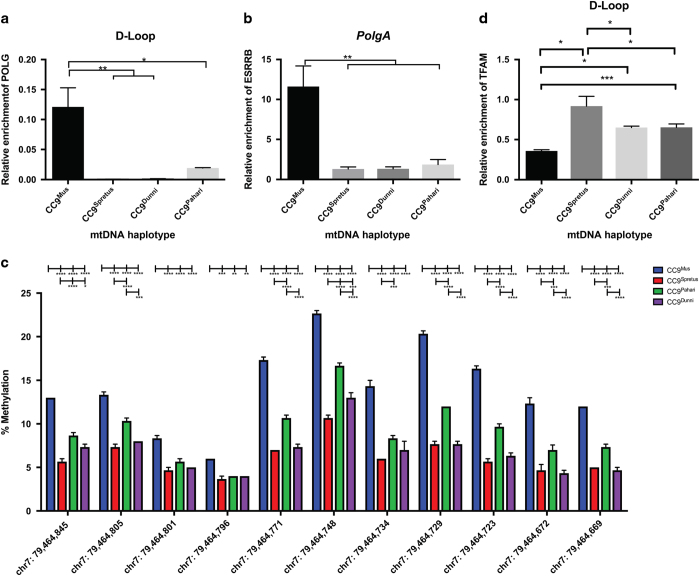
The levels of enrichment for POLGA, ESRRB and TFAM and DNA methylation at exon 2 of *PolgA*. (**a**) Levels of enrichment for *PolgA* in the O_H_ in the D-loop region of the mitochondrial genome following ChIP using antibodies specific to POLGA and real-time PCR across the O_H_ region; (**b**) levels of enrichment for ESRRB within the CpG island of *PolgA* as determined by ChIP using an anti-ESRRB antibody and real-time PCR across the region of interest in *PolgA*. (**c**) % Methylation of *PolgA* at exon 2 for CC9^mus^, CC9^spretus^, CC9^pahari^ and CC9^dunni^ cells. Pyrosequencing was performed for 11 CpGs found on mouse *PolgA* exon 2 (chr7: 79 464 669–79 464 845). Primers were designed using the mouse reference sequence from UCSC Genome Browser Dec. 2011 (GRCm38/mm10) Assembly. (**d**) Levels of enrichment for TFAM in the D-loop region of the mitochondrial genome following ChIP using an anti-TFAM antibody and real-time PCR. Data are expressed as mean±S.E.M. Statistical analysis was performed using two-way ANOVA followed by Tukey’s multiple comparisons test. *P<0.05; **P<0.01; ***P<0.001; ****P<0.0001.

To determine the degree of POLGA affinity for each of the mtDNA haplotypes, using a chromatin immunoprecipitation (ChIP) assay, we assessed its levels of enrichment at its primary binding site in the origin of replication of the heavy strand (O_H_) in the D-loop where mtDNA replication is initiated. In undifferentiated cells, the levels of POLGA enrichment were greater in the CC9^mus^ cells (Figure 4a). There was a similar outcome for the enrichment of EsRRB (Figure 4b), a key regulator of pluripotency that binds just upstream of the DNA methylated CpG island in exon 2 of *PolgA.* This suggests that the interaction of a key regulator of pluripotency and the mtDNA-specific replication factor are more tightly regulated in undifferentiated CC9^mus^ ES cells, and more efficient at maintaining the mtDNA set point and restricting precocious differentiation than for the other haplotypes. When we assessed the levels of DNA methylation in the CpG island at exon 2 of *PolgA* by pyrosequencing, undifferentiated CC9^mus^ cells exhibited higher levels of DNA methylation at each of the 11 sites compared with the other divergent lines (Figure 4c). Again, using a ChIP assay, there was discordant binding affinity for TFAM, the mitochondrial transcription factor that initiates mtDNA replication (Figure 4d). Likewise, there was discordant binding affinity in the coding genes, namely the *ATPase6* (Supplementary Figure S1A), cytochrome B (Supplementary Figure S1B), *Cox1* (Supplementary Figure S1C) and *Nd1* (Supplementary Figure S1D) genes, where TFAM likely acts as a packaging protein.^24^

### DNA methylation induced by the divergent mtDNA haplotypes in the CC9 chromosomal genome

**Figure 5:**
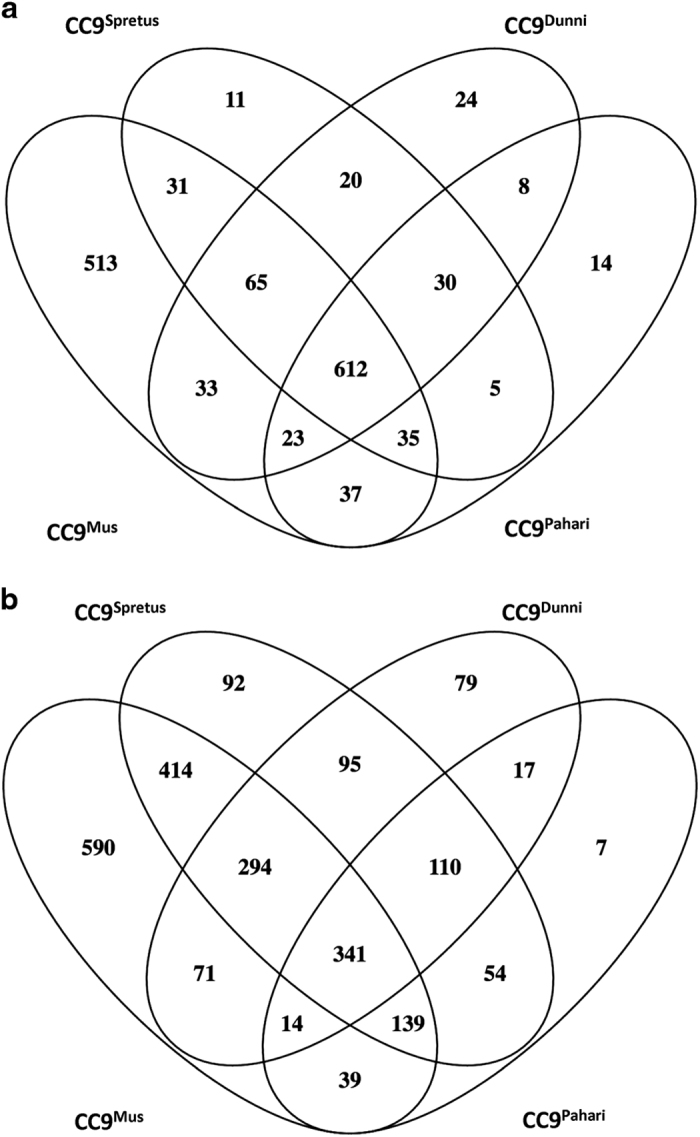
Analysis of hyper- and hypomethylated genes for each of the divergent mtDNA ES cell lines. The methylation status for each of the lines was determined by MeDIP array using an antibody to 5mC, and data were collected using the Agilent CytoGenomics Analytic software (v2.9). (**a**) Hypermethylated genes; (**b**) hypomethylated genes.

As there were different patterns of neural gene expression and discordant patterns of binding affinity for POLGA and TFAM for each mtDNA haplotype, we assessed DNA methylation patterns amongst the ES cell lines using the 2×105K CpG microarray. We identified 8351 probes, which were assigned to 4243 loci of which 3552 were known genes. The CC9^mus^ ES cells were the most distinctive with 513 hypermethylated (Figure 5a) and 590 hypomethylated (Figure 5b) genes specific to this line. The CC9^spretus^ cells had 11 hypermethylated (Figure 5a) and 92 hypomethylated (Figure 5b) genes, the CC9^dunni^ line 24 hypermethylated (Figure 5a) and 79 hypomethylated (Figure 5b) genes, and the CC9^pahari^ line 14 hypermethylated (Figure 5a) and 7 hypomethylated (Figure 5b) genes.

Following assignment to DAVID for functional annotation clustering,^25^ 5 of the 513 hypermethylated CC9^mus^ genes (Supplementary Table S3) were associated with the mitochondrion, 122 with transcriptional regulation and 40 with neuronal differentiation and development. Of the hypomethylated CC9^mus^ genes, 53 genes were associated with the mitochondrion (Supplementary Table S4), 91 with transcriptional regulation and 12 with neuronal differentiation. These DNA methylation patterns are likely to influence mitochondrial respiration, cellular function and differentiation. The 11 hypermethylated CC9^spretus^ genes mostly affected zinc finger proteins, which regulate DNA- and protein-binding functions whilst the 92 hypomethylated genes affected nuclear function and DNA binding. Most of the hypermethylated CC9^dunni^ genes affected the regulation of transcription and RNA metabolic processing. Amongst the 79 hypomethylated CC9^dunni^ genes, 19 were associated with microtubules and cytoskeleton, 8 with cell cycle and 7 with RNA processing.

### Modulation of the regulators of DNA methylation in divergent ES cell lines

**Figure 6:**
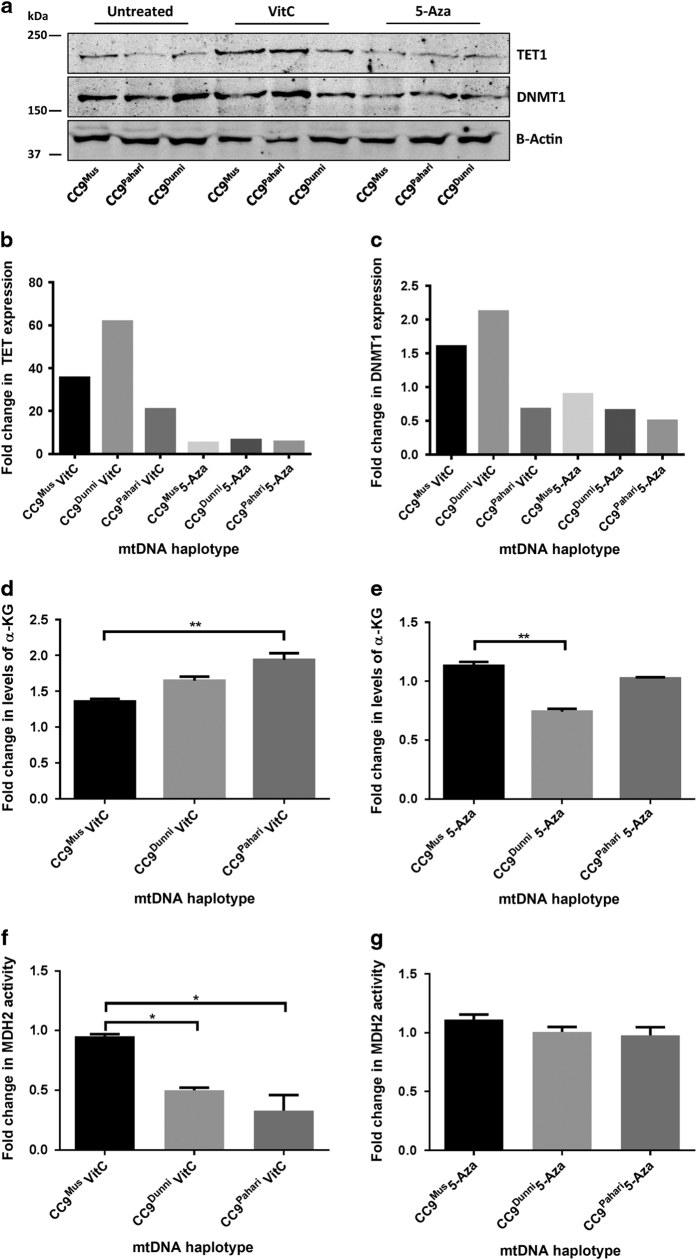
VitC increases TET1 expression and modulates *α*-KG levels and MDH2 activity. (**a**) CC9^mus^, CC9^dunni^ and CC9^pahari^ ES cells were treated with VitC and 5-Aza, and TET1 and DNMT1 protein levels were measured by western blot. Actin protein levels were used to confirm equivalent loading and the precision plus all blue protein marker (Biorad, Gladesville, NSW, Australia) was used used to determine protein size. TET1 (**b**) and DNMT1 (**c**) expression was normalised to actin and expressed as the fold change in expression compared to vehicle-treated cells. *α*-KG levels increased as a result of VitC treatment (**d**) but not 5-Aza (**e**) whilst MDH2 activity decreased as a result of VitC treatment (**f**) but 5-Aza had no effect (**g**) when compared to non-treated cells from the same line with values represented as fold change to non-treated cells. *P<0.05; **P<0.01.

To determine whether the extensive hypermethylation patterns observed in the divergent ES cells could be modulated by DNA demethylation agents, we cultured CC9^mus^, CC9^dunni^ and CC9^pahari^ ES cells in the presence of 5-Azacytidine (5-Aza) and vitamin C (VitC) for 48 and 72 h, respectively. 5-Aza modulates DNA methyltransferase 1 (DNMT1) and, therefore, inhibits DNA methylation during cell division,^26^ whilst VitC acts on TET1 to promote the conversion of 5mC to 5hmC to demethylate DNA.^27^ The addition of VitC greatly increased the expression of TET1 in each of the lines (range=~20- to >60-fold) whilst 5-Aza marginally affected TET1 activity (Figures 6a and b). Addition of 5-Aza upregulated DNMT1 expression slightly in the three lines though there were also increases with VitC (Figures 6a and c). This is likely to be a response to the inhibition of DNMT1 from interacting within promoter regions by 5-Aza and VitC being unable to inhibit DNMT1 activity. *α*-Ketoglutarate (*α*-KG) is a product of the tricarboxylic acid (TCA) cycle and is a cofactor in the conversion of 5mC to 5hmC to demethylate DNA.^28^ VitC increased levels of *α*-KG (Figure 6d) whilst 5-Aza reduced levels in CC9^dunni^ and CC9^pahari^ cells (Figure 6e). We also examined mitochondrial malate dehydrogenase 2 (MDH2) activity, as MDH2 occurs before *α*-KG in the TCA cycle, and observed a corresponding fold change decrease in CC9^dunni^ and CC9^pahari^ cells with VitC treatment (Figure 6f) but there was no change for 5-Aza (Figure 6g). These results suggest that mtDNA haplotypes modulate the TCA cycle to regulate global DNA methylation patterns.

### Modulation of chromosomal gene expression by addition of VitC and 5-Aza to divergent ES cells

**Figure 7:**
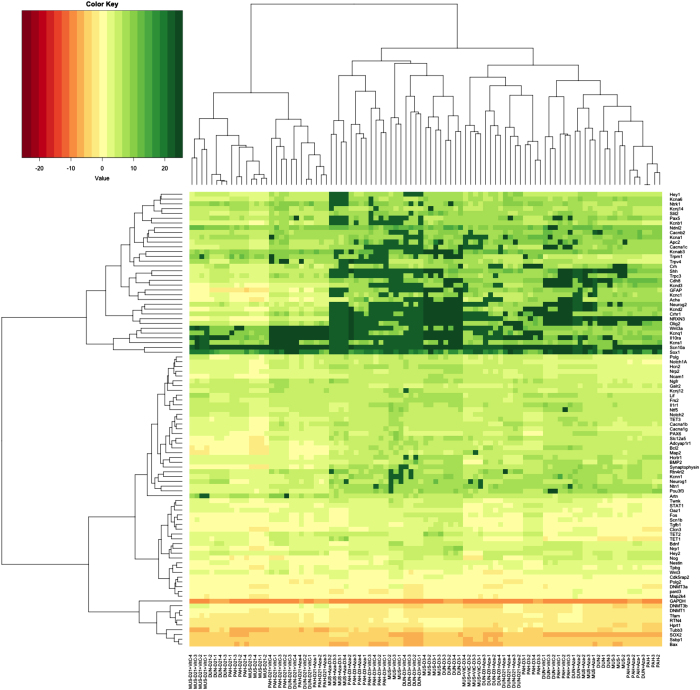
Heatmap of gene expression profiles across divergent mtDNA ES lines. CC9^mus^, CC9^dunni^ and CC9^pahari^ ES cells, both untreated and post DNA demethylation treatment (VitC and 5-Aza), were induced to differentiate into neural lineages over 21 days and the differentiation status was compared at days 0, 3 and 21. *n*=3 for all undifferentiated samples and 4 for all differentiated samples. Samples were clustered in columns and gene targets were clustered in rows with the euclidean distance clustering method. The heatmap was plotted based on the Ct values normalised to the Ct value of the housekeeping gene 18S rRNA. The plot was generated using the HTqPCR package.

To determine whether the changes to the modulators of DNA methylation induced by 5-Aza and VitC affected chromosomal gene expression patterns, we induced cells cultured with 5-Aza and VitC to undergo neural differentiation. Using a Fluidigm array, we analysed undifferentiated, and days 3 and 21 differentiated cells for neural-specific markers associated with neurogenesis, neuronal differentiation, endpoint neural differentiation, neuronal ion channels and neuronal signal transduction that had exhibited either hypermethylation or hypomethylation. We also analysed regulators of DNA methylation, and mtDNA transcription and replication (Supplementary Tables S5; Figure 7).

For the regulators of DNA methylation following VitC treatment, there were overall decreases in gene expression on day 0 except for the CC9^dunni^ population, increases on day 3 and decreases on day 21 (Supplementary Table S5; Figure 7). Similar patterns were observed for 5-Aza (Supplementary Table S6), although day 21 CC9^mus^ cells did not survive. These outcomes reflect the translation of the transcripts into protein (cf Figure 6; Supplementary Tables S5 and S6). For the mtDNA transcription and replication factors, each of the lines behaved differently with no clear patterns. For the markers of neurogenesis, overall, VitC induced decreases in expression levels in undifferentiated cells and increases on day 3 whilst, on day 21, levels were downregulated in CC9^mus^ and CC9^pahari^ cells but were equally up- and downregulated in CC9^dunni^ cells. 5-Aza treatment only induced an overall change (downregulation) in CC9^mus^ and CC9^pahari^ cells for the neurogenesis markers on day 0, whilst, on day 3, all three lines upregulated expression. On day 21, there was overall downregulation. Consequently, there appears to be effective regulation of the neurogenesis markers with anticipated upregulation on day 3 and downregulation on day 21. For neuronal differentiation, both treatments induced upregulation of gene expression on day 3 but variable outcomes for day 21 with CC9^mus^ and CC9^pahari^ cells downregulating expression after VitC with similar outcomes for CC9^pahari^ and CC9^dunni^ cells after 5-Aza treatment, which is anticipated for genes associated with differentiation. However, neither treatment induced overall increased levels of expression for genes of endpoint markers, neuronal ion channels or neuronal signal transduction. Consequently, resetting DNA methylation patterns in undifferentiated mtDNA divergent ES cells did not enhance differentiation potential.

## Discussion

Whilst mtDNA haplotypes are associated with predisposition to disease,^18,19,20,21^ adaptation to environments^16,17^ and fertility,^23,29^ it has not been apparent how these outcomes are induced. Previously, we had shown that mtDNA haplotypes can modulate neural gene expression patterns in ES cells and their propensity to form beating cardiomyocytes.^15^ Here, we show a mechanistic approach where mtDNA haplotypes modulate key regulators of DNA methylation, DNMT1 and TET1. We further show the release of *α*-KG from the TCA cycle, which is a cofactor in the conversion of 5mC to 5hmC by TET1.^28^ Nuclear–mitochondrial compatibility is important for establishing functional electron transfer chains.^30^ However, alternate metabolic pathways, such as the TCA cycle, appear to be more affected when challenged by VitC-induced DNA demethylation. To this extent, whilst each of the lines produced greater levels of *α*-KG and TET1, the more divergent combination of CC9^pahari^ cells produced twofold more *α*-KG.

The mtDNA set point is important for establishing and maintaining pluripotency in undifferentiated cells and for regulating mtDNA copy number during differentiation.^9,12,14,31^ The interactions between the nucleus and the mitochondrial genome likely modulate the mtDNA set point to accommodate the requirements of both genetic compartments of the cell,^14^ which suggests that the potential to differentiate into certain lineages is regulated by this interaction. Indeed, CC9^mus^ cells were the most efficient at regulating pluripotency through the increased affinity of ESRRB for *PolgA* in undifferentiated cells and synchronising increases in mtDNA copy number with stage-specific changes in gene expression during differentiation. Nevertheless, the use of DNA demethylation agents prevented the cells from completing differentiation, suggesting that having already established the set point had enabled the two genomes to establish their mechanisms of interaction.

During postimplantation development when cells retain their undifferentiated status, mtDNA replication is restricted to replenishing copy number, as is the case in ES cells.^9^ However, mtDNA replication is tested between E7.5 and E10.5 when *PolgA*^32^ and *Tfam*^33^ homozygous knockout mice die *in* *utero*, which is equivalent to the key mtDNA turnover events identified in ES cells.^9^ Not only is this likely to be important for testing whether the mtDNA set point has been adequately established, but it enables the mitochondrial and chromosomal genomes to determine how they will collectively function. This involves the chromosomal genome-regulating levels of mtDNA copy number to maintain the mtDNA set point to mediate pluripotency and to respond to cues to differentiate, as shown by the differing affinities for the enrichment of *M. musculus*-encoded TFAM, POLGA and ESRRB. In turn, the mitochondrial genome influences the chromosomal genome by regulating OXPHOS activity through the degree of compatibility of the proteins that is encodes for the electron transfer chain. If cells build less functional electron transfer chains, negative feedback could result in reduced TCA cycle activity, as indicated by decreased MDH2 activity following VitC, that would result in increased levels of *α*-KG and promote the conversion of 5mC to 5hmC,^28^ as modulated by VitC.^27^ Similarly, mtDNA-depleted cells have been shown to modulate histone acetylation marks through the TCA cycle as they restore mtDNA copy number.^34^ Consequently, the initial stages of development involve a genomic ‘tug-of-war’ that establishes the most advantageous genomic conditions for cells to function effectively, which will affect their ability to complete differentiation as evidenced by the varying degrees of success for the mtDNA divergent ES cells.

In establishing the appropriate interactions between the mitochondrial and nuclear genomes, it is likely that a trade-off takes place to promote phenotype.^35^ Evolutionary trade-offs exist between reproductive capacity and energetic expense in birds,^36^ where females who invest in larger oocytes exhibit larger reproductive organs, a larger body mass and higher resting metabolic rate,^36^ which makes them less fit for other activities. In commercial pigs, mtDNA haplotypes influence litter size and reproductive efficiencies over multiple generations^23^ but those with low fertility are maintained as they offer other benefits such as better meat quality. Likewise, cows that have enhanced growth and carcase traits often have poor fertility.^37,38,39^

Adaptation to an oocyte’s mtDNA background has important implications for assisted reproductive technologies such as somatic cell nuclear transfer (transfer of a donor cell into an enucleated recipient oocyte),^40^ pronuclear transfer (transfer of pronuclei from a fertilised oocyte)^41^ and metaphase II spindle transfer (transfer of the spindle from a mature oocyte).^42^ Somatic cell nuclear transfer can result in perturbed DNA methylation patterns,^43^ initially thought to arise from incomplete reprogramming. However, our work suggests that the divergence between the donor cell, carrying chromosomal DNA, and the recipient oocyte, harbouring mtDNA, may be too great. This has important implications for the use of somatic cell nuclear transfer for the generation of new super breeds of livestock where, for example, chromosomes carrying specific genetic markers for sought-after phenotypic traits, such as enhanced milk or meat quality, could be introduced into oocytes with mitochondrial haplotypes associated with increased fertility.^44^ Similar problems arise when metaphase II spindle transfer or pronuclear transfer are used to prevent the transmission of mutant mtDNA. Appropriate mtDNA matching would ensure that the resultant cells, tissues and organs had compatible mitochondrial and chromosomal genomes, especially as key imprinting events take place very early during development.^45^

In conclusion, using an ES cell model that tests one set of chromosomes against several divergent mtDNA haplotypes, we have shown that mtDNA haplotypes influence chromosomal gene expression by modulating DNA methylation. Each mtDNA divergent ES cell line established its own DNA methylation profile that could be altered by DNA demethylation agents, which resulted in fold changes in levels of *α*-KG and perturbed chromosomal gene expression profiles. Furthermore, each of the lines differentially replicated its mtDNA in a specific manner, which was associated with precocious gene expression profiles in the more divergent haplotypes during differentiation. These outcomes could have serious implications for those using nuclear transfer to prevent the transmission of mtDNA disease and account for the disorders associated with somatic cell nuclear transfer.

## Materials and methods

Additional materials and methods appear in Supplementary Information.

### Mouse ES cell culture and differentiation

*M. musculus* CC9.3.1 ES cells that were previously reconstructed to harbour *M. musculus* mtDNA (CC9^mus^) and mtDNA from more divergent subspecies *M. spretus* (CC9^spretus^), *M. terricolor* (CC9^dunni^) and *M. pahari* (CC9^pahari^) were cultured and differentiated, with minor modifications, as previously described^15^ (Supplementary Information). For DNA demethylation experiments, undifferentiated ES cells cultured in feeder-free conditions were treated with VitC (Sigma-Aldrich, Castle Hill, NSW, Australia) at a final concentration of 100 *μ*g/ml or 5-Aza (Sigma-Aldrich) at a final concentration of 0.5 *μ*M for 72 and 48 h, respectively.

### DNA and RNA extraction and cDNA synthesis

Total DNA and RNA were extracted using the DNeasy Blood and Tissue Kit and RNeasy Mini Kit (both Qiagen, Valencia, CA, USA), respectively, according to the manufacturer’s protocol. DNA samples were treated with RNase solution (Qiagen) and Proteinase K solution (Qiagen) at 65 °C for 10 min while RNA samples were treated with DNase I (Qiagen) for 20 min. cDNA was synthesised from 1 *μ*g of total RNA using oligo(dT) primers and the Superscript III First-Strand synthesis system (Thermo Fisher, Scoresby, VIC, Australia), according to the manufacturer’s instructions.

### Next-generation sequencing of mitochondrial genomes

Next-generation sequencing of complete mitochondrial genomes was performed on amplified long PCR products. Long PCR reactions were prepared, as described in ref. 46 (Supplementary Information) and PCR products purified using the QIAquick PCR Purification Kit (Qiagen), according to the manufacturer’s protocol. Purified amplicon pairs were combined at equal concentrations, and amplicon libraries were generated using the recommended workflow procedures from the Ion Fragment Library Kit and Ion Xpress Template kit using 318 chips and run on an Ion Torrent PGM (all Thermo Fisher).

DNA fragments were mapped to a mouse mtDNA reference genome (accession: AP013031), using the CLC Genomics Workbench v7.5.1 (Qiagen), to assemble each mtDNA sequence. The voting strategy was used for base-pair calling. The accession numbers for the mtDNA sequences are KY018919 (*M. musculus*), KY018920 (*M. dunni*), KY018921 (*M. spretus*) and KY038052 (*M. pahari*).

### Phylogenetic analysis

Model testing was performed using CLC Genomics Workbench, as described in Tsai *et al*.^23^ Using the GTR model,^47,48^ a Maximum Likelihood tree was created with 1000 bootstrap replicates to show the relationship between the different mtDNA haplotypes. Further details are available in the Supplementary Information.

### Evolutionary analyses

Evolutionary analyses were conducted in MEGA6.^49^ The complete mtDNA sequences for each *Mus* species and *Rattus norvegicus* (NC_001662.2) were aligned using ClustalW followed by model testing. The General Time Reversible model^47^ had the lowest Bayesian Information Criterion scores, and was, therefore, selected.^48^ A Maximum Likelihood phylogenetic tree was constructed by applying the Neighbor-Joining method to a matrix of pairwise distances estimated using the Maximum Composite Likelihood approach. A discrete Gamma distribution was used to model evolutionary rate differences amongst sites (five categories (+G, parameter=0.3734)). The tree was drawn to scale, with branch lengths measured by the number of substitutions per site. The tree was supported by 1000 bootstrap replicates. Estimation of divergence time was performed using the RelTime method.^50^ Calibration constraints were based on the *R. norvegicus* and *M. musculus* split of 8–12 Mya.^51^

### Pyrosequencing of exon 2 of *PolgA*

Pyrosequencing assays were designed using the PyroMark Assay Design Software (Version 2.0.1, Qiagen). A unit of 500 ng DNA samples were converted using the Epitect Bisulphite Conversion Kit (Qiagen), as per the manufacturer’s protocol. The region of interest was amplified by PCR using PyroMark PCR Kit (Qiagen) and prepared for pyrosequencing, as described in Supplementary Table S7. Pyrosequencing was performed on a PyroMark 24 Pyrosequencing System (Qiagen), as per the manufacturer’s instructions. Data were analysed on the PyroMark Q24 software to determine the % methylation values for each CpG site in the sample.

### Immunoprecipitation of methylated DNA

Immunoprecipitation of methylated DNA (MeDIP) was performed, as previously described.^14,31^ Briefly, 3 *μ*g of the sonicated DNA was immunoprecipitated with 2 *μ*g of either 5mC (Active Motif, Carlsbad, CA, USA) or 5hmC (Active Motif) at 4 °C overnight, and the immunoprecipitated DNA was purified using the Qiagen PCR Purification Kit (Qiagen). Further details are available in the Supplementary Information.

### Chromatin immunoprecipitation

ChIP was performed as previously described.^15^ Cells were crosslinked then sonicated to fragment chromatin to an average size of 200–800 bp. Chromatin from 1×10^6^ cells was immunoprecipitated with Protein G Dynabeads and an anti-POLGA antibody (G-6, Santa Cruz Biotechnology, Inc., Dallas, TX, USA), or anti-TFAM antibody (Santa Cruz Biotechnology, Inc.), or anti-ESRRB antibody (H6705, R&D Systems, Minneapolis, MN, USA). Crosslinks in immunoprecipitated samples were reversed and pulled-down samples purified using the QIAquick PCR Purification Kit (Qiagen). Further details are available in the Supplementary Information.

### Real-time PCR to assess mtDNA copy number, mRNA expression, ChIP and MeDIP

All real-time PCR (quantitative PCR, qPCR) reactions were performed on a RotorGene 3000 real-time PCR machine (Corbett Research, Mortlake, NSW, Australia). The number of mtDNA copies/cell were quantified against external standards for β-actin and mtDNA, as previously described in Kelly *et al*.^15^ All primers used are listed in Supplementary Table S7. mRNA expression levels were determined by the ΔΔCt method, as described in Kelly *et al*.,^15^ all primers used are listed in Supplementary Table S7. Real-time PCR was performed on MeDIP and ChIP samples using primers amplifying gene regions of interest (Supplementary Table S7) to determine enrichment against input samples, as described in Kelly *et al*.^15^

### CpG array (MeDIP array)

A unit of 400 ng of input and 150 ng 5mC-containing DNA samples purified from MeDIP, as described above, were used for each CpG microarray (Agilent, Mulgrave, VIC, Australia). Input DNA was Cy3-labelled and the methylated DNA fraction Cy5-labelled using the Agilent SureTag DNA labelling kit for 4 h at 37 °C. Samples were column-purified and combined with Cot-1 DNA, Deionised Formamide, CGH blocking agent and HI-RPM hybridisation buffer (Agilent). Samples were hybridised on mouse (105K) CpG island microarrays (015279- Agilent) for 40 h at 67 °C. Arrays were washed according to the Agilent CGH protocol, immediately scanned on an Agilent microarray scanner and processed using Agilent Feature extraction software version 11.0.1.1.

The data were processed using the Agilent CytoGenomics Analytic software (v2.9). Once the data were obtained, data filtering was done on SPSS v24.0 (IBM CORP, St. Leonards, NSW, Australia). To identify the differentially methylated probes and genes, we analysed the data by using a cutoff of fourfold differential methylation ratios (log2 ratio >+2 or <−2) between the samples. We then calculated the mean differential DNA methylation ratios for each of the groups and combined the data with another cutoff obtained from the *Z*-scores for each of the probes identified. As the *Z*-scores reflect the relative distance of the log ratios of a probe to the Gaussian distributions of other probes with similar melting temperatures on the array, we further filtered the data using *Z*-scores >+5 or<−5 as cutoffs.

### *α*-KG quantification

*α*-KG was quantified using the *α*-Ketoglutarate Assay Kit (Sigma-Aldrich), according to the manufacturer’s instructions. In all, 2×10^6^ cells were homogenised in *α*-KG buffer, the samples were mixed with a coupled enzyme, and the resulting fluorometric product measured using a FLUOstar Optima plate reader (BMG Labtech, Mornington, VIC, Australia) at Ex/Em=544/590 nm. The amount of *α*-KG per sample in nmol (Ay) was determined from the *α*-KG standard curve using the equation: Ay=(corrected absorbance−(*y*−intercept)/slope). All experimental samples were run as replicates and all samples and standards measured in duplicate. Results are expressed as fold change in *α*-KG levels compared to untreated cells of each line.

### MDH2 assay

MDH2 activity was determined following the manufacturer’s protocol (Abcam, 119693). Cells were lysed in extraction buffer, 50 *μ*g of protein were bound to antibody capture plates, enzyme activity buffer containing a reagent dye was added and absorbance at 450 nm was recorded every 30 s for 30 min on a FLUOStar Optima plate reader (BMG Labtech). Enzyme activity was calculated by: *U*=(*r*_A_×*V*_cuvette_)/(*l*×*ξ*×*V*_sample_×*ρ*), where *r*_A_=rate of absorbance change; *V*_cuvette_=volume of the solution; *l*=optical path length; *ξ*=extinction coefficient; *V*_sample_=volume sample; *ρ*=mass concentration of material. The extinction coefficient of the reagent dye was 37/mM/cm. Results are expressed as fold change in MDH2 activity compared to untreated cells of each line.

### Fluidigm array and analysis

Pre-amplification was performed on cDNA samples, as described in the Gene Expression Preamp with Fluidigm Preamp MasterMix (Fluidigm, San Francisco, CA, USA) and Taqman Assays Quick Reference PN 68000133 RevC protocol. In all, 96 Taqman assays were selected, as listed in Supplementary Table S8, and pooled with C1 DNA suspension buffer to produce a final concentration for each assay of 180 nM. A volume of 1.25 *μ*l of each cDNA sample and a non-template control underwent pre-amplification for 14 cycles with 3.75 *μ*l of pooled assays and Taqman PreAmp Master Mix (Life Technologies), according to the manufacturer’s instructions.

Assays and samples were combined in a 96.96 Dynamic array Integrated Fluidic Circuit (IFC) plate, according to the Fluidigm 96.96 Real-Time PCR Workflow Quick Reference PN 6800088 protocol. Using the IFC controller HX, 5 *μ*l of each pre-amplified sample was loaded as duplicates into each sample inlet and 5 *μ*l of each Taqman assay (10×) was loaded into the assay inlet of the plate. Gene expression was performed according to the Biomark GE 96.96 Standard v2 Protocol. Data were exported using the Fluidigm Real-Time PCR analysis software (v4.1.1). Differentially expressed genes were analysed using the HTqPCR package (version 1.26). The normalisation of ‘deltaCt’ and the Limma method were used.

### Genomic data sets

The accession numbers for the MeDIP array data sets reported in this paper are deposited as NCBI GEO: GSE94918 (http://www.ncbi.nlm.nih.gov/geo/). The mtDNA next-generation sequencing data are deposited at GenBank (https://www.ncbi.nlm.nih.gov/genbank/). The respective accession numbers are KY018919 (*M. musculus*), KY018920 (*M. dunni*), KY018921 (*M. spretus*) and KY038052 (*M. pahari*).

## Additional infomation

**Publisher’s note:** Springer Nature remains neutral with regard to jurisdictional claims in published maps and institutional affiliations.
