# Supplementary material for: Mitochondrial DNA haplotypes induce differential patterns of DNA methylation that result in differential chromosomal gene expression patterns
Source: Cell Death Discov. 2017 Sep 11;3:17062–. doi: 10.1038/cddiscovery.2017.62 (PMC5592988; doi:10.1038/cddiscovery.2017.62)
Supplement: Supplementary Information [file cddiscovery201762-s1.docx]

**Supplementary Information**

**Title: Mitochondrial DNA haplotypes induce differential patterns of DNA methylation that result in differential chromosomal gene expression patterns.**

**Authors: William Lee ^1,2^*, Xin Sun ^1,2^*, Te-Sha Tsai ^1,2^*, Jacqueline Johnson ^1,2^*, Jodee A. Gould^3^, Daniel J Garama^2,4^, Daniel J. Gough ^2,4^, Matthew McKenzie ^1,2^, Ian Trounce ^5^, and Justin C. St. John ^1,2^.**

**^1^ Centre for Genetic Diseases, Hudson Institute of Medical Research, 27-31 Wright Street, Clayton, Vic 3168, AUSTRALIA.**

**^2^ Department of Molecular and Translational Science, Monash University, 27-31 Wright Street, Clayton, Vic 3168, AUSTRALIA.**

**^3^ Medical Genomic Facility, Monash Health Translational Precinct, 27-31 Wright Street, Clayton, Vic 3168, AUSTRALIA.**

**^4^ Centre for Cancer Research, Hudson Institute of Medical Research, 27-31 Wright Street, Clayton, Vic 3168, AUSTRALIA.**

**^5^ Centre for Eye Research Australia, Ophthalmology, University of Melbourne Department of Surgery, Vic., Australia.**

Correspondence: Justin St. John, Centre for Genetic Diseases, Hudson Institute of Medical Research, Clayton, Vic 3168, AUSTRALIA, +61 3 8572 2678, +61 3 9594 7416, justin.stjohn@hudson.org.au

*These authors all contributed equally to this work.

**Materials and Methods**

**Mouse Embryonic Stem (ES) cell culture and differentiation**

*Mus musculus* mtDNA (CC9^mus^), *Mus spretus* (CC9^spretus^), *Mus terricolor* (CC9^dunni^) and *Mus pahari* (CC9^pahari^) cells were cultured on feeder cells composed of mitomycin C-treated embryonic fibroblasts. All cell lines were cultured in ES cell media consisting of DMEM, 15% FBS, 2mM GlutaMax, 1% MEM nonessential amino acids, 0.5% penicillin-streptomycin, 0.1mM β-mercaptoethanol (all from ThermoFisher Scientific, Waltham, MA, USA) and 1000U/ml of ESGRO Leukemia inhibitory factor (LIF) (Merck Millipore, Bayswater, VIC, Australia) under conditions of 37°C and 5% CO_2_. Cells were transferred to feeder-free conditions prior to experimental use and differentiation.

For differentiation, undifferentiated ES cells, both untreated and post-treatment, were dissociated and plated onto 0.1% gelatin-coated tissue culture plates at a density of 1.5 - 2 × 10^4^/cm^2^ in ES cell, no LIF, media. After 1 day, media was changed to N2B27 medium, comprising DMEM/F12 supplemented with 2% B27, 1% N2 (all from ThermoFisher Scientific) and 10ng/ml bFGF (Merck Millipore). Medium was renewed every 2 days. At day 7, the cultures were dissociated using Accutase (Sigma-Aldrich) and plated out at 5 x 10^4^/ cm^2^ on an ornithine/laminin substrate in Neurobasal media with 2% B27 (both from ThermoFisher Scientific) and 10ng/ml BDNF (Merck Millipore). Medium was changed every three days and cells were differentiated for 21 days.

**Next generation sequencing of mitochondrial genomes**

Next generation sequencing of whole mitochondrial genomes was performed on amplified long PCR products. Each reaction consisted of 50 ng total DNA, 1x High Fidelity PCR buffer, 100 mM MgSO4, 1 mM dNTPs (Bioline, London, UK), 1U of Platinum Taq High Fidelity (Invitrogen, Carlsbad, CA, USA) and 10μM each of the forward and reverse primer (A forward CCGTGCTACCTAAACACCTTATC and A reverse CGTCCGTACCATCATCCAATTA; B forward CCCTTCATCCTTCTCTCCCTAT and B reverse GTGGGATCCCTTGAGTTACTTC). Reaction conditions were 94°C for 2:00, 94°C for 0:15, 57°C for 0:30, 68°C for 10:00 (34 cycles), 68°C for 10:00, held at 4°C. PCR products were purified using QIAquick PCR Purification Kit (Qiagen), according to the manufacturer’s protocol. Purified amplicon pairs generated from long PCR were combined at equal concentrations, prior to generation of the libraries. Amplicon libraries were generated using the recommended workflow procedures from the Ion Fragment Library Kit and Ion Xpress™ Template kit using 318 chips and run on an Ion Torrent PGM (all ThermoFisher Scientific).

**Phylogenetic analysis**

CLC Genomics Workbench was used to perform model testing to identify the best model for the Maximum Likelihood phylogenetic tree construction. Four different statistical analyses were used: hierarchical likelihood ratio test, Bayesian information criterion, Minimum theoretical information criterion and Minimum corrected theoretical information criterion. The models tested were Jukes-Cantor, Felsenstein 81, Kimura 80, HKY and GTR (also known as the REV model). The model deemed the best by most statistical tests was GTR ^1, 2^. A Maximum Likelihood tree was created with 1000 bootstrap replicates to show the relationship between the different mtDNA haplotypes.

**Evolutionary analyses**

Evolutionary analyses were conducted in MEGA6 ^3^. The complete mtDNA sequences of *Rattus norvegicus* (NC_001662.2), *Mus musculus* (KY018919)*, Mus Dunni* (KY018920)*, Mus spretus* (KY018921) *and Mus pahari* (KY038052) were aligned using ClustalW followed by model testing to determine the best model for phylogenetic tree construction. The General Time Reversible model ^1^ had the lowest BIC scores (Bayesian Information Criterion), and was therefore selected ^2^. The Maximum Likelihood phylogenetic tree was constructed by applying the Neighbor-Joining method to a matrix of pairwise distances estimated using the Maximum Composite Likelihood (MCL) approach. A discrete Gamma distribution was used to model evolutionary rate differences amongst sites (5 categories (+G, parameter = 0.3734)). The tree was drawn to scale, with branch lengths measured by the number of substitutions per site. The tree was supported by 1000 bootstrap replicates. Estimation of divergence time was performed using the RelTime method ^4^. Calibration constraints were based on the *Rattus norvegicus* and *Mus musculus* split of 8-12 Mya ^5^.

**Pyro-sequencing of exon 2 of POLGA**

All pyrosequencing assays were designed using the PyroMark Assay Design Software (Version 2.0.1, Qiagen). Briefly, a 200 bp reference sequence was entered into the software to design primers covering the target CpG sites. DNA samples were converted using the Epitect Bisulphite Conversion Kit (Qiagen). 500 ng of genomic DNA was converted overnight in a total volume of 140 µL, according to the manufacturer’s instructions. Converted DNA was isolated on columns and stored at -20° C.

The assay region containing the CpG target sites was amplified by PCR using a biotin labelled, HPLC purified primer (MousePolG1_RB: TTCCCTCTACCAAACAAACCT) and a standard sequencing grade primer (MousePolG1_F: GGGGGTAATTTGGATTAGTATTTT). All PCR amplifications were performed with the PyroMark PCR Kit (Qiagen). Amplification reactions consisted of 12.5 µL PyroMark Mastermix, 2.5 µL Coral Load, 1 µL each of 5 µM forward and reverse primers, 2 µL of bisulphite converted DNA template and 6 µL of ddH_2_O. Thermocycling conditions consisted of 15 minutes at 95 °C, followed by 45 cycles of 30 seconds at 95 °C, 30 seconds at 56 °C and 30 seconds at 72 °C and a final extension step of 10 minutes at 72 °C. All amplicons were visualised on 2% agarose gels to confirm quality and determine concentration.

PCR products were then bound to Streptavidin Sepharose High Performance beads (GE Healthcare Life Sciences, Parramatta, NSW, Australia). The immobilized PCR products were denatured and washed using proprietary solutions (Qiagen) on the Pyrosequencing Vacuum Prep Tool (Qiagen) to isolate single stranded DNA. The beads were transferred to an optically clear, 24 well sequencing plate in 0.3 µM of pyrosequencing primer (MousePolG1_S1: GTAATTTGGATTAGTATTTT; MousePolG1_S2: TTATTGGAGGTTTAATTGTTT; MousePolG1_S4: GGGTTTTGGTGTT). Primer annealing to the single-stranded template was performed by heating to 80 °C followed by cooling to room temperature. Pyrosequencing was performed on a PyroMark 24 Pyrosequencing System (Qiagen), as per the manufacturer’s instructions. Data were analysed on the PyroMark Q24 software to determine the % methylation values for each CpG site in the sample.

**Immunoprecipitation of methylated DNA**

Immunoprecipitation of methylated DNA (MeDIP) was performed, as previously described ^6, 7^. Briefly, 3 μg of the sonicated DNA, which ranged between 200 to 1000 bp in size, was denatured at 95°C for 10 minutes and immunoprecipitated with 2 μg of either 5-methylcytosine (5mC; Active Motif, USA) or 5-hydroxymethylcytosine (5hmC; Active Motif) with 20 μl of Protein G Dynabeads (ThermoFisher Scientific) in 500 μl of IP buffer (10mM Na-phosphate, pH 7.0, 140mM NaCl, 0.05% Triton X-100) at 4°C overnight. Samples were washed three times with 700 μl IP buffer and DNA was eluted using 250 μl of Proteinase K digestion buffer (5mM Tris, 1mM EDTA, pH8.0, 0.05% SDS) with 7 μl of 10mg/ml Proteinase K (Qiagen) at 50°C for 3 hours. The immunoprecipitated DNA was then purified using the Qiagen PCR Purification Kit (Qiagen).

**Chromatin immunoprecipitation**

Chromatin immunoprecipitation (ChIP) was performed, as previously described ^8^. Briefly, cells were freshly collected and cross-linked with 1% formaldehyde (Sigma-Aldrich) for 10 min and quenched with 125 mM glycine (Sigma-Aldrich) for 5 min. Cross-linked cells were lysed with SDS lysis buffer (50 mM Tris–HCl (pH 8), 10 mM EDTA, and 1 % SDS) on ice for 15 min and sonicated to fragment chromatin to an average size of 200 to 800 bp. Chromatin from 1 x 10^6^ cells was immunoprecipitated with Protein G Dynabeads and anti-POLGA antibody (G-6, Santa Cruz Biotechnology, Inc., CA, USA), or anti-TFAM antibody (Santa Cruz Biotechnology, Inc.), or anti-ESRRB antibody (H6705, R&D Systems, MN, USA) in ChIP dilution buffer (0.01% SDS, 1.1% Triton X­100, 1.2 mM EDTA, 16.7 mM Tris–HCl ((pH 8.1)), and 167 mM NaCl). Immunoprecipitated samples were washed twice in low salt washing buffer (20 mM Tris–HCl ((pH 8.1)), 2 mM EDTA, 150 mM NaCl, 0.1% SDS, and 1% Triton X­100); high salt washing buffer (20 mM Tris–HCl (pH 8.1), 2 mM EDTA, 500 mM, 0.1% SDS, and 1% Triton X­100); and TE buffer (10 mM Tris–HCl (pH 8) and 1 mM EDTA). The samples were then eluted and reverse cross-linked by incubating in elution buffer (0.1 M NaHCO3 and 1% SDS) with 200 mM NaCl and 10 μl of Proteinase K at 65°C for 16 h. Pull-down samples were purified using the QIAquick PCR Purification Kit (Qiagen). Enrichment relative to the corresponding input sample (%) was analysed using qPCR with primers listed in Table S7.

**Supplementary Data**


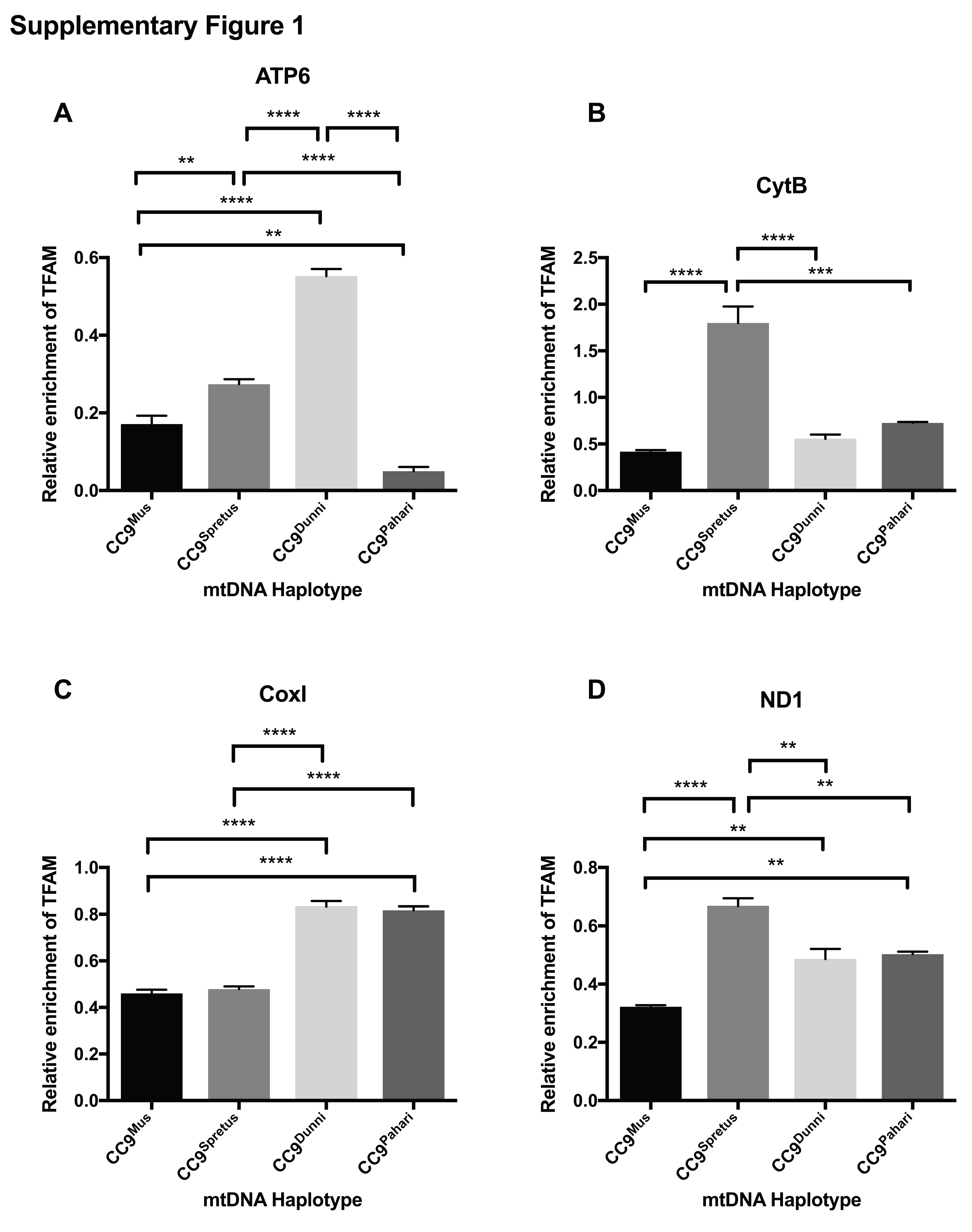


**Figure S1: Levels of enrichment for TFAM within the coding and non-coding regions of the mitochondrial genome for the divergent ES cell lines.** Levels of enrichment for TFAM were determined by ChIP using an anti-TFAM antibody and real time PCR across A) *Atp6*; B) *Cytb*; C) *CoxI*; and D) *Nd1*. * = P<0.05; ** = P<0.01; *** = P<0.001: **** = P<0.0001.

**Table S2. Comparisons of amino acids that are unique to the mitochondrial genomes of each of the divergent mtDNA cell lines.**

|  | **CC9^mus^** | **CC9^dunni^** | **CC9^spretus^** | **CC9^pahari^** |
| --- | --- | --- | --- | --- |
| **NADH1** | S157N, I175L, T261M | L165F, L176I, L251M, N258S, Y317H | V11L, F19Y, I39V, M57I, H93Y, I175V | I4V, L46F, M68I, V87I, T167N, L251I, T261L, A263T, M318V |
| **NADH2** | P151S, I213A | A7T, F11S, M27L, S36N, I41V, I45M, T57M, G92S, M156T, L157M, T160M, M206A, M225L, T242M, I244T, N310S, T315I, T320I, M332I, L343M | F11L, M85V | A7M, V18I, T24S, V75I, T83M, G92H, L95I, M97L, T98A, S103C, F113H, P147N, M156I, T160I, M164T, I210L, A218M, I230L, A239I, M271V, M278I, A279T, L281M, M284I, M304L, M313L, M314Y, T315L, P322L, L324F, M325T, F326L, S327P, T328L, A330T |
| **COXI** | Y403F | M483A | None | T177S, T408M, S486T, A488S, Y510F |
| **COXII** | None | None | M21T | M75L |
| **ATPase8** | S53L | P34T, T43M, V47T | S41L | T32N, S41A, T44I |
| **ATPase6** | I50V | N183S | None | M14V, A22T, K35E, T69A, T115L |
| **COXIII** | I168M, V248I | L45I, I51V, I168L | N38S | I40T, T41I, I175V |
| **NADH3** | I5T, T89I | I11T, T16A, S85T, | None | Y4F, I7M, F8V, I9T, I11S, T16I, V20I, S85N, M88T, T89A, A103M, T107M |
| **NADH4L** | M37V | T4V, S13A, T47I | T4A, T47A, V81I | S3Y, F6L, S13I, S45A, S55N, T62I |
| **NADH4** | F6L, V100I, I171V, T188A, I449T | L9F, N51T, N54I, N88S, N89L, V90I, I107T, L176F, M177T, F181L, T183A, V351I, T382A, M383I, I398M, L444I | S19N, T37V, T40S, I104A, T185A, F380Y, M459I | S19Q, T25M, T37I, L39F, N48S, K50I, S57F, N88K, N89S, V90T, A112S, T173S, T182S, T183S, H184N, T185P, T188Y, S240G, I248V, D251N, K255E, M310S, F380L, M396I, I398V, L434M |
| **NADH5** | I69V, I82T, L214M, N272F, V302I, N476S, I479V, P606L | L16I, H29Y, T38M, L49I, H56Y, N57S, I69M, E75K, K77T, I82L, T268A, N271S, N361S, I477V, M513T, A516T, K540M, L544P, T561V, ins608W | I45L, M71V, I211L, N272Y, F485L, N514S, M589L, S590T, I596T | I3V, L9I, L10S, L14M, I19M, L20A, M23A, S24I, N25Y, L26P, H29N, I30S, T36A, T38S, I45F, L49M, N57Y, F182L, M193I, L198F, F203L, N205D, N207S, D208del, N209del, L210del, V261I, T268S, L283I, M319I, A351G, T363M, V374I, K434N, S442T, M451I, F463M, F485Y, T489M, M509T, A516I, Y519F, S520T, S521P, L525Q, F528Y, S531L, I536M, M539K, L544H, K547N, T548M, T551N, L553M, T561L, L569I, M573F, T575K, L576M, T577L, I593V, N605S |
| **NADH6** | V102L, Y104C, V115I | G90S, I97V, V105M, N107G, Y108N, Y109N, V131I | V7I, F101V, G119A | V7A, S10L, V14T, L87M, L89F, I97L, F101L, V105L, L106F, G113E, N116D, D118E, G119S, V128I |
| **CYTB** | I39V, M102L, F238I | L192M | A23T, F238L | I232A, T241I, I323T, I327L, I334V |

**Table S5. Fold change in gene expression in undifferentiated and differentiating CC9 cells harbouring *Mus musculus*, *pahari* and *dunni* mtDNA following initial culture with VitC.** Arrows indicate up and down regulation, respectively.

| **VitC** | **No. of genes** | **CC9^mus^**  **Day 0** | **CC9^mus^**  **Day 3** | **CC9^mus^ Day 21** | **CC9^pahari^**  **Day 0** | **CC9^pahari^ Day 3** | **CC9^pahari^**  **Day 21** | **CC9^dunni^**  **Day 0** | **CC9^dunni^ Day 3** | **CC9^dunni^ Day 21** |
| --- | --- | --- | --- | --- | --- | --- | --- | --- | --- | --- |
| **Regulators of DNA methylation** | 6 | 6🡻 | 6🡹 | 6🡻 | 6🡻 | 5🡹  1🡻 | 5🡻 | - | 5🡹 | 2🡹  4🡻 |
| **mtDNA transcription and replication factors** | 5 | 5🡻 | 5🡹 | 5🡻 | 4🡻 | 3🡹 | 2 🡻 | 2🡻 | 4🡹 | 3🡹  2🡻 |
| **Neurogenesis** | 31 | 1🡹  26🡻 | 13🡹  1🡻 | 29🡻 | 4🡹  23🡻 | 12🡹  7🡻 | 6🡹  21🡻 | 3🡹  8🡻 | 12🡹  2🡻 | 9🡹  9🡻 |
| **Neuronal differentiation** | 16 | 12🡻 | 8🡹  3🡻 | 14🡻 | 12🡻 | 7🡹  8🡻 | 15🡻 | 2🡹  3🡻 | 5🡹  2🡻 | 5🡹  6🡻 |
| **Endpoint neuronal**  **differentiation** | 3 | 2🡻 | 2 🡹 | 3🡻 | 2🡻 | 1🡹  1🡻 | 3🡻 | - | - | 2🡻 |
| **Neuronal ion channel** | 24 | 2🡹  16🡻 | 10🡹  2🡻 | 16🡻 | 1🡹  17🡻 | 4🡹  10🡻 | 20🡻 | 1🡹  5🡻 | 8🡹  1🡻 | 4🡹  11🡻 |
| **Neuronal signal transduction** | 7 | 2🡹  3🡻 | 6🡹  1🡻 | 7🡻 | 2🡹  4🡻 | 2🡹  5🡻 | 1🡹  5🡻 | 2🡹 | 2🡹  4🡻 | 1🡹  5🡻 |

**Table S6. Fold change in gene expression in undifferentiated and differentiating CC9 cells harbouring *Mus musculus*, *pahari* and *dunni* mtDNA following initial culture with 5-Aza.** Arrows indicate up and down regulation, respectively.

| **5-Aza** | **No. of genes** | **CC9^mus^**  **Day 0** | **CC9^mus^**  **Day 3** | **CC9^mus^ Day 21** | **CC9^pahari^**  **Day 0** | **CC9^pahari^ Day 3** | **CC9^pahari^ Day 21** | **CC9^dunni^**  **Day 0** | **CC9^dunni^ Day 3** | **CC9^dunni^ Day 21** |
| --- | --- | --- | --- | --- | --- | --- | --- | --- | --- | --- |
| **Regulators of DNA methylation** | 6 | 6 🡻 | 2🡹  3🡻 | Na | 6 🡻 | 3🡹  1🡻 | 6🡻 | - | 5🡹 | 2🡹  3🡻 |
| **mtDNA transcription and replication factors** | 5 | 5 🡻 | 2🡹 | Na | 4 🡻 | 4 🡹 | 5🡻 | - | 5🡹 | 2🡹  2🡻 |
| **Neurogenesis** | 31 | 4🡹  12🡻 | 18🡹  4🡻 | Na | 1🡹  15🡻 | 15🡹  5🡻 | 2 🡹  24🡻 | 3 🡹  4 🡻 | 17 🡹 | 4 🡹  21🡻 |
| **Neuronal**  **Differentiation** | 16 | 6🡻 | 7🡹  3🡻 | Na | 3🡹  11🡻 | 5🡹  7🡻 | 1🡹  13🡻 | 1🡻 | 12🡹  1🡻 | 3🡹  8🡻 |
| **Endpoint neuronal**  **differentiation** | 3 | - | - | Na | 2 🡻 | 1🡻 | 3🡻 | - | 1🡹 | 3🡻 |
| **Neuronal ion channel** | 24 | 3 🡹  11🡻 | 5🡹  5🡻 | Na | 2🡹  10🡻 | 5🡹  5 🡻 | 18🡻 | 2🡻 | 8🡹 | 2🡹  19🡻 |
| **Neuronal signal transduction** | 7 | 1🡹  4 🡻 | 3🡹  3🡻 | Na | 2🡹  3🡻 | 2🡹  4🡻 | 6🡻 | 1🡻 | 4🡹  1🡻 | 1🡹  6🡻 |

**Table S7. Primer pairs for real time PCR, ChIP and pyrosequencing.**

| **Gene region** | **Sequence** | **Size (bp)** | **Tm (**°C**)** |
| --- | --- | --- | --- |
| **mtDNA copy number** | | | |
| *ActB* | F: CCCTACAGTGCTGTGGGTTT  R: GAGACATGCAAGGAGTGCAA | 205 | 57 |
| *tRNA/Cox1* | F: CAGTCTAATGCTTACTCAGC  R: GGGCAGTTACGATAACATTG | 273 | 56 |
| **Neuronal gene expression** | | | |
| *Gfap* | F: TCCTGGAACAGCAAAACAAG  R: CAGCCTCAGGTTGGTTTCAT | 54 | 224 |
| *Pax6* | F: GAGAGGACCCATTATCCAGATG  R: CCATTTGGCCCTTCGATTAGA | 56 | 108 |
| Sox1 | F: GGCCGAGTGGAAGGTCAT  R: ACTTGTAATCCGGGTGTTCCT | 56 | 101 |
| *Tubb3* | F: TGAGGCCTCCTCTCACAAGT  R: GGCCTGAATAGGTGTCCAAA | 56 | 105 |
| *Ncam* | F: TTCCTGTGTCAAGTGGCAGGAGAT  R: AGATCTTCACGTTGACAGTGGCCT | 60 | 229 |
| *Nestin* | F: CTACCAGGAGCGCGTGGC  R: TCCACAGCCAGCTGGAACTT | 60 | 219 |
| *Musashi* | F: CACGGTGGAAGATGTGAAACA  R: TCGCTCTCAAACGTGACAAATC | 56 | 120 |
| *Map2* | F: AAAGGCCCGCGTAGATCAC  R: GGGATTCGAGCAGGTTGATG | 57 | 122 |
| *Synaptophysin* | F: TGCAGAACAAGTACCGAGAG  R: CTGTCTCCTTAAACACGAACC | 56 | 297 |
| *rRNA18S* | F: GTAACCCGTTGAACCCCATT  R: CCATCCAATCGGTAGTAGCG | 56 | 151 |
| **TFAM ChIP** | | | |
| D-loop | F: CTCAACATAGCCGTCAAGGC  R: ACCAAACCTTTGTGTTTATGGG | 435 | 57 |
| *CytB* | F: ACCCGCCCCATCCAACATTT  R: GGGATGGCTGATAGGAGGTT | 339 | 60 |
| *Cox1* | F: GCCCACCACATATTCACAGTAGG  R: GGCGAAGTGGGCTTTTGCTC | 381 | 60 |
| *Atp6* | F: ACTTCCTTCCACAAGGAACTCC  R: TGGTAGCTGTTGGTGGGCTAAT | 192 | 58 |
| *Nd1* | F: ACGAGCCGTAGCCCAAACAA  R: GGGCCGGCTGCGTATTCTAC | 258 | 57 |
| **POLG ChIP** | | | |
| D-loop-O_H_ | F: CGGGTCTAATCAGCCCATGA  R: TGAGTAGCATTTATGTCTAACAAGC | 205 | 57 |
| **EsRRB ChIP** | | | |
| *Polg* | F: TTCTGTTACGCCTCCAACAA  R: GGGTTAGGGCCACTCGAC | 273 | 56 |
| **Pyrosequencing (*denotes single strand primers)** | | | |
| MousePolG1_Rbiotin  MousePolG1_F | TTCCCTCTACCAAACAAACCT  GGGGGTAATTTGGATTAGTATTTT | 285 | 56 |
| MousePolG1_S1* | GTAATTTGGATTAGTATTTT | 74 | 80 |
| MousePolG1_S2* | TTATTGGAGGTTTAATTGTTT | 80 | 80 |
| MousePolG1_S4* | GGGTTTTGGTGTT | 69 | 80 |

**Table S8. Taqman assays used for the Fluidigm Array.**

| **Assay name** | **Type** | **Assay ID** |
| --- | --- | --- |
| NRXN3 | Neurogenesis | Mm04279482_m1 |
| Ntrk1 | Neurogenesis | Mm01219406_m1 |
| Notch1 | Neuronal signal transduction | Mm00435249_m1 |
| BMP2 | Neuronal differentiation | Mm01340178_m1 |
| PAX6 | Neuronal differentiation | Mm00443081_m1 |
| SOX2 | Neuronal differentiation | Mm03053810_s1 |
| Bcl2 | Neurogenesis | Mm00477631_m1 |
| Map2k4 | Neurogenesis | Mm00436508_m1 |
| Cacna1b | Neuronal ion channel | Mm01333678_m1 |
| Il1r1 | Neurogenesis | Mm00434237_m1 |
| Notch2 | Neuronal signal transduction | Mm00803077_m1 |
| Tgfb1 | Neurogenesis | Mm00441724_m1 |
| Bax | Neurogenesis | Mm00432050_m1 |
| STAT1 | Neurogenesis | Mm00439531_m1 |
| pard3 | Neurogenesis | Mm00473929_m1 |
| Cacna1c | Neuronal ion channel | Mm01188822_m1 |
| Cacna1g | Neuronal ion channel | Mm00486572_m1 |
| RTN4 | Neuronal differentiation | Mm00445861_m1 |
| Hcn2 | Neuronal ion channel | Mm00468538_m1 |
| Scn10a | Neuronal ion channel | Mm00501467_m1 |
| Kcnd3 | Neuronal ion channel | Mm01302126_m1 |
| Frs2 | Neurogenesis | Mm00769591_m1 |
| Cacnb2 | Neuronal ion channel | Mm00659092_m1 |
| Kcnc1 | Neuronal ion channel | Mm00657708_m1 |
| Trpc3 | Neuronal ion channel | Mm00444690_m1 |
| Cdk5rap2 | Neuronal differentiation | Mm00524401_m1 |
| Apc2 | Neurogenesis | Mm00478649_m1 |
| Kcnq1 | Neuronal ion channel | Mm00434640_m1 |
| Kcnj12 | Neuronal ion channel | Mm00440058_s1 |
| Kcnb1 | Neuronal ion channel | Mm00492791_m1 |
| Scn1b | Neuronal ion channel | Mm00441210_m1 |
| Kcnn1 | Neuronal ion channel | Mm01349167_m1 |
| Ntn1 | Neurogenesis | Mm00500896_m1 |
| Kcns1 | Neuronal ion channel | Mm00492824_m1 |
| Bdnf | Neuronal differentiation | Mm04230607_s1 |
| Rtn4rl2 | Neurogenesis | Mm01336368_g1 |
| Kcna1 | Neuronal ion channel | Mm00439977_s1 |
| Ngfr | Neurogenesis | Mm00446296_m1 |
| Neurog2 | Neuronal differentiation | Mm00437603_g1 |
| Pou3f3 | Neuronal differentiation | Mm00843792_s1 |
| Adcyap1r1 | Neurogenesis | Mm00431683_m1 |
| Tpbg | Neurogenesis | Mm00495741_s1 |
| Ntf5 | Neurogenesis | Mm01701591_m1 |
| Crhr1 | Neurogenesis | Mm00432670_m1 |
| Slc12a5 | Neuronal ion channel | Mm00803929_m1 |
| Trpm1 | Neuronal ion channel | Mm00450619_m1 |
| Il10ra | Neurogenesis | Mm00434151_m1 |
| Neurog1 | Neuronal differentiation | Mm00440466_s1 |
| Artn | Neurogenesis | Mm00507845_m1 |
| Kcnj14 | Neuronal ion channel | Mm01194051_g1 |
| Galr2 | Neurogenesis | Mm00726392_s1 |
| Cdh8 | Neurogenesis | Mm00483238_m1 |
| Ndnl2 | Neurogenesis | Mm00480974_s1 |
| Kcna6 | Neuronal ion channel | Mm00496625_s1 |
| Ache | Neurogenesis | Mm00477274_g1 |
| Nrp1 | Neurogenesis | Mm00435379_m1 |
| Kcnd2 | Neuronal ion channel | Mm01161732_m1 |
| Hcrtr1 | Neurogenesis | Mm01185776_m1 |
| Slit2 | Neurogenesis | Mm00662153_m1 |
| Fos | Neurogenesis | Mm00487425_m1 |
| Crh | Neurogenesis | Mm01293920_s1 |
| Hey2 | Neuronal signal transduction | Mm00469280_m1 |
| Kcnab3 | Neuronal ion channel | Mm01337143_m1 |
| Nog | Neuronal differentiation | Mm01297833_s1 |
| Pax5 | Neuronal differentiation | Mm00435501_m1 |
| Nrp2 | Neurogenesis | Mm00803099_m1 |
| Clcn3 | Neuronal ion channel | Mm01348786_m1 |
| Hey1 | Neuronal signal transduction | Mm00468865_m1 |
| Lif | Neurogenesis | Mm00434762_g1 |
| Trpv4 | Neuronal ion channel | Mm00499025_m1 |
| TET1 | Methylation regulators | Mm01169087_m1 |
| TET2 | Methylation regulators | Mm00524395_m1 |
| TET3 | Methylation regulators | Mm00805756_m1 |
| DNMT1 | Methylation regulators | Mm01151063_m1 |
| DNMT3a | Methylation regulators | Mm00432881_m1 |
| DNMT3b | Methylation regulators | Mm01240113_m1 |
| Ncam1 | Neuronal differentiation | Mm01149710_m1 |
| Nestin | Neuronal differentiation | Mm00450205_m1 |
| Sox1 | Neuronal differentiation | Mm00486299_s1 |
| Tubb3 | Neuronal differentiation | Mm00727586_s1 |
| GFAP | End marker | Mm01253033_m1 |
| Synaptophysin | End marker | Mm00436850_m1 |
| Shh | Neuronal signal transduction | Mm00436528_m1 |
| Wnt3a | Neuronal signal transduction | Mm00437337_m1 |
| Wnt3 | Neuronal signal transduction | Mm00437336_m1 |
| Olig2 | Neuronal differentiation | Mm01210556_m1 |
| Map2 | End marker | Mm00485231_m1 |
| Polg | mtDNA Replication Factor | Mm00450527_m1 |
| Polg2 | mtDNA Replication Factor | Mm00450166_m1 |
| Tfam | mtDNA Transcription Factor | Mm00447485_m1 |
| Ssbp1 (MtSSB) | mtDNA Replication Factor | Mm01131763_g1 |
| Peo1 Twink) | mtDNA Replication Factor | Mm00467928_m1 |
| Hprt1 | Housekeeping gene | Mm00446968_m1 |
| GAPDH | Housekeeping gene | Mm03302249_g1 |
| 18S | Housekeeping gene | Mm04277571_s1 |
| Oaz1 | Housekeeping gene | Mm01611061_g1 |

**References**

1. Yang Z. Estimating the pattern of nucleotide substitution. *J Mol Evol* 1994, **39**(1)**:** 105-111.

2. Nei M, Kumar, S. . *Molecular Evolution and Phylogenetics*. Oxford University Press: New York, 2000.

3. Tamura K, Stecher G, Peterson D, Filipski A, Kumar S. MEGA6: Molecular Evolutionary Genetics Analysis version 6.0. *Mol Biol Evol* 2013, **30**(12)**:** 2725-2729.

4. Tamura K, Battistuzzi FU, Billing-Ross P, Murillo O, Filipski A, Kumar S. Estimating divergence times in large molecular phylogenies. *Proc Natl Acad Sci U S A* 2012, **109**(47)**:** 19333-19338.

5. Catzeflis FM, Aguilar JP, Jaeger JJ. Muroid rodents: Phylogeny and evolution. *Trends Ecol Evol* 1992, **7**(4)**:** 122-126.

6. Lee W, Johnson J, Gough DJ, Donoghue J, Cagnone GLM, Vaghjiani V*, et al.* Mitochondrial DNA copy number is regulated by DNA Methylation and demethylation of POLGA in stem and cancer cells and their differentiated progeny. *Cell Death and Disease* 2015, **6**(e1664).

7. Lee WTY, Cain JE, Cuddihy A, Johnson J, Dickinson A, Yeung KY*, et al.* Mitochondrial DNA plasticity is an essential inducer of tumorigenesis. *Cell Death Discovery* 2016(2)**:** 16016.

8. Kelly RD, Rodda AE, Dickinson A, Mahmud A, Nefzger CM, Lee W*, et al.* Mitochondrial DNA haplotypes define gene expression patterns in pluripotent and differentiating embryonic stem cells. *Stem Cells* 2013, **31**(4)**:** 703-716.
